# Every Step Counts: Reported Walking Ability as a Predictor of All-Cause Mortality

**DOI:** 10.1093/geroni/igaf122.786

**Published:** 2025-12-31

**Authors:** Keisha Lovence, Shanshan Yao, Ziling Mao, Elsa Strotmeyer, Eleanor Simonsick, Anne Newman

**Affiliations:** University of Pittsburgh, Pittsburgh, Pennsylvania, United States; University of Michigan, Ann Arbor, Michigan, United States; University of Pittsburgh, Pittsburgh, Pennsylvania, United States; University of Pittsburgh, Pittsburgh, Pennsylvania, United States; National Institute on Aging, Baltimore, Maryland, United States; University of Pittsburgh, Pittsburgh, Pennsylvania, United States

## Abstract

Evaluation of mortality risk with respect to physical function has focused on limitation. Here, we evaluated all-cause mortality risk in relation to increments of capacity in mobility intact older adults using the Walking Ability Index (WAI). In the Health, Aging and Body Composition study (Health ABC, N = 3075, aged 70-79), self-reported ease/difficulty walking ¼ mile and 1 mile, scored zero (worst) to nine (best) was assessed. Per study eligibility requirements, the WAI ranged from 4-9 at baseline. This analysis includes 2812 participants (57% White; 49% men; aged 73.6±2.9 years). Mortality over 15 years was ascertained from death certificates and proxy reports. Analyses used Kaplan-Meier plots, and Cox regression adjusting for demographic, lifestyle, and health factors. For baseline WAI, 673 (24%) scored 4-6, 855 (30%) scored 7-8, and 1284 (46%) scored 9. During a follow-up of 15 years, 1578 (56%) participants died. Kaplan-Meier analysis demonstrated significant association between higher WAI scores and better survival (log-rank test p < 0.001). A 1-point higher WAI score corresponded to a lower adjusted mortality risk (HR = 0.88, 95% CI: 0.85–0.92). A dose-response relationship was observed, with scoring 9 on the WAI associated with lowest mortality risk (0.61[0.46–0.81]) referent to scoring 4. Findings strongly indicate that attention to functional capacity provides important information about the likelihood of longevity and argues for including capacity assessments within research and health care settings involving older adults. Future work should interrogate relationships between self-reported walking ability and other aging outcomes.

